# Lacrimal gland removal impairs sexual behavior in mice

**DOI:** 10.3389/fnana.2014.00101

**Published:** 2014-09-25

**Authors:** Rosa Maria Cavaliere, Filippo Ghirardi, Roberto Tirindelli

**Affiliations:** Department of Neuroscience, University of ParmaParma, Italy

**Keywords:** ESP1, lacrimal glands, vomeronasal, pheromones, lordosis behavior

## Abstract

Exocrine gland-secreting peptides (ESPs) are a protein family involved in the pheromonal communication of rodents. ESP1 is a lacrimal peptide synthesized by the extraorbital glands of males of specific mouse strains that modulates the sexual behavior in females. Reportedly, BALB/c males, that produce high level of ESP1 in the tear fluid, were shown to enhance the lordosis behavior in C57BL/6 females during mating. In contrast, C57BL/6 and ICR males, both unable to express ESP1, failed to modulate this sexual behavior. Nonetheless, ICR males did become competent to enhance lordosis behavior in C57BL/6 females providing these were pre-exposed to ESP1. To exclude any strain differences, here, we investigated the pheromonal role of the extraorbital glands and indirectly of ESP1 in animals of the same strain. This was performed by applying the lordosis experimental paradigm in BALB/c mice before and after the surgical removal of these glands in males. The excision of the extraorbital glands reduced but did not abolish the production of ESP1 in the lacrimal fluid of BALB/c mice. An immunological analysis on soluble extracts of the glands that drain into the conjunctival sac revealed that the intraorbital glands (ILGs) are also responsible for the production of ESP1. The removal of both the extra and ILGs completely eliminated the tear secretion of ESP1. Extraorbital gland-deficient BALB/c mice were still able to induce lordosis behavior in sexually receptive females. In contrast, males with the removal of both the extra and ILGs failed to enhance lordosis behavior in females. Unexpectedly, C57BL/6 males did improve this sexual performance in BALB/c females. However, an analysis of the tear fluid of C57BL/6 males revealed low but detectable levels of ESP1. Overall, our study highlights the relevance of the orbital glands in modulating reproductive behavior and the sensitivity of the vomeronasal system to detect trace amount of ESP1.

## Introduction

Mammals produce chemically different types of pheromones to mediate social behavior (Tirindelli et al., 2009). Small volatile pheromones diffuse in the air and mediate long distance communication. In contrast, peptide pheromones are dropped on the ground or even they remain on the body surface, allowing signal transmission between conspecifics but exclusively by direct contact.

In the mouse, peptide pheromones are reportedly excreted in the urine, vaginal smear and also through the tear fluid (Mucignat-Caretta et al., 1995; Hurst et al., 2001; Briand et al., 2004; Leinders-Zufall et al., 2004; Brennan and Kendrick, 2006; Chamero et al., 2007). The pheromonal components of salivary and lacrimal secretion include molecules belonging to a multigenic protein family named exocrine gland-secreting peptides (ESPs; Kimoto et al., 2005, 2007). ESP1 is a sex specific pheromone that is specifically expressed by the extraorbital glands (ELGs) of males of some mouse strains (including BALB/c) but not in others (including C57BL/6 and Slc:ICR) and released into tear fluids (Kimoto et al., 2005, 2007). ESP1 is detected by the chemosensory neurons of the vomeronasal organ of Jacobson (Kimoto et al., 2005), a secondary olfactory structure that lies underneath the nasal septum and opens into the anterior part of the nasal cavity. The vomeronasal organ expresses two groups of G- protein coupled receptors, namely V1Rs and V2Rs that sense both volatile and non volatile pheromones (Dulac and Axel, 1995; Herrada and Dulac, 1997; Matsunami and Buck, 1997; Ryba and Tirindelli, 1997). ESP1 is recognized by a specific V2R and the information is conveyed to brain centers where it elicits neuroendocrine and behavioral responses (Haga et al., 2010). In particular, ESP1 seems to modulate the lordosis behavior (Haga et al., 2010) which represents a sexual response of female mice to allow successful mating and it is characterized by a reflexive posture upon male mounting (Keller et al., 2006).

Haga et al. recorded the sexual behavior of a C57BL/6 receptive female mouse introduced into a cage housing either an ESP1-secreting male (BALB/c) or a male that does not produce ESP1 (C57BL/6). They found that females introduced to a BALB/c male showed a significant increase in lordosis response compared to females mated to a C57BL/6 male. To firmly establish the pheromonal role of ESP1, they also demonstrated that BALB/c females, pre-exposed to ESP1, displayed an increased lordosis behavior when challenged with a stud male of a non producing ESP1 strain as Slc:ICR (Haga et al., 2010).

These results provide strong evidence that ESP1 effectively enhances female sexual behavior. However, these studies did not entirely clarified whether the behavioral responses elicited by ESP1 would be influenced by the different mouse strains that were employed in these experiments. Mice are in fact able to selectively discriminate conspecifics of different strains (Leinders-Zufall et al., 2004; Cheetham et al., 2009; Kaur et al., 2014). Thus, we wanted to investigate if the lordosis behavior was not affected when tests were carried out on mice of the same strain (BALB/c). To accomplish this task, it was firstly necessary to remove the ELGs of male BALB/c mice in order to abolish the ESP1 production. Unexpectedly, we found that the excision of ELGs resulted in a reduced but still detectable level of ESP1 in the tear fluid. Analysis of the lacrimal glands that drain into the conjunctival sac showed that two small lateral glands, the intraorbital glands (ILGs), were responsible for the remaining production of ESP1. Removal of both ELGs and ILGs completely abolished any residual ESP1 immunoreactivity in the tear fluid. BALB/c males deprived of the ELGs and ILGs but not of ELGs alone failed to enhance lordosis behavior in BALB/c females. These findings establish the pheromonal role of the orbital glands in modulating sexual behavior most likely through the action of ESP1 and possibly other lacrimal secretions.

## Materials and methods

### Animals

Mice of the BALB/c and C57BL/6 strain were purchased from Harlan and bred in our animal house. Mice were maintained on a 12 h dark, 12 h light cycle. For both immunological and behavioral studies, 2–4 month old mice were employed.

The experiments comply with the Principles of Animal Care of the National Institutes of Health and with the current law of the European Union and Italy. The present project was approved by the Ethical Committee of the University of Parma: approval ID: 17/14, date: 27/03/14.

### Surgery

For ovariectomy in BALB/c and C57BL/6, 1 month old female mice were anesthetized with a solution (0.1 ml/ 10 g weight) of xylazine (1 mg/ml) and ketamine (10 mg/ml).

Mice were placed ventrally and the dorsal surgical area was shaved and disinfected. A short dorsal midline skin and abdominal muscle incision was made halfway between the last dorsal rib and the base of the tail. The ovary and the oviduct were exteriorized. A sterile ligature was placed around the oviduct and the ovary was removed. The surgical plan was then closed with silk sutures.

For lacrimal gland surgery in BALB/c male mice, animals were anesthetized as above described. The main extraorbital lacrimal gland in the mouse is located just rostrally to the parotid gland and lies on the masseter muscle. The intraorbital gland is located rostrally to the extraorbital gland in proximity to the lateral commissure and lies on the supraorbital branch of the superficial temporal artery. The two glands are connected by the lacrimal duct that drains the fluid into the conjunctival sac. A skin incision was made between the lateral commissure of the eye and ear. After dissection of the subcutaneous tissue, the lacrimal gland was identified and exposed. By using forceps, the gland was easily separated from the parotid gland and exteriorized. The lacrimal duct was identified and closed with non-reabsorbable suture and the gland was removed. The lacrimal duct was then followed up to the junction with the intraorbital gland that, in turn, was removed making sure not to damage the superficial temporal vein (STV) and artery. Animals were left to recover for 2 weeks during which the eyes were lubricated and maintained aseptic with eye drops containing antibiotics and Celluvisc^©^.

### Behavioral tests

For the *Courtship behavior test*, after ovariectomy, oestrus was induced in sexually naïve females by estradiol benzoate treatment (0.02 mg s.c. 24 h and 48 h before test) followed by a single progesterone injection (0.3 mg s.c.) 4 h before testing. Female mice were then caged individually and always tested in one assay.

Stud males were selected by training to mount oestrous females until they exhibit a number of mounts greater than 20 in 30 min. Each sexually experienced male mouse was isolated for 48 h before test without replacing the bedding.

In each experiment, a naive oestrus female was introduced into a cage housing an isolated stud male and their behavior was videotaped for 30 min. All experiments were performed in 17 × 25 cm Plexiglas transparent cages that allow the ventral view of the videotaping. The experiments were always carried out at the same time of day (3–5 p.m.).

For the analysis, the total numbers of stud male mounts, female lordosis behavior towards mounts and the intromission behavior towards females was scored according to Haga et al. (2010). Briefly, mount was defined as a stud male climbed onto a female for copulation. Lordosis response was positive when female, with all four paws on the cage floor, elevated the hind region while arching the back and tilting the head upwards. Intromission was defined as a repetitive male pelvic thrust during copulation. The experimenter analyzing behavior was blind to the experimental condition of the animals.

For the *Emergence* test, mice were placed inside a small dark box (15 × 14 × 12 cm), containing one exit door. The box was located in the middle of a large cage (33 × 55 × 18 cm). After 5 min habituation, the door was opened and the latency before emerging from the box, the number of walks out of the box and the time spent inside the box were measured in a 5 min trial. Locomotor activity was also observed during the period outside the box. The experiments were always carried out at the same time of day (3–5 p.m.).

Behavioral performances were analyzed by using “The Observer” software.

Statistical analysis was performed by ANOVA with Student’s *t*-test and by Levene test (variance homogeneity) on SPSS 20.0 platform.

### Sample collection and western blotting

Tear fluids were collected twice a day for 2 weeks, washing the eye with 15 μl of a solution containing 5 mM sodium phosphate pH 7.4. The lacrimal fluids were then pooled, lyophilized and re-supended in water before electrophoresis.

Glandular soluble extract were obtained by homogenizing the collected glands in 10 mM Tris-HCl pH 7.5. The suspension was then ultracentrifuged for 30 min at 1000 g. The aqueous phase was harvested and the protein concentration was measured (Bio-Rad assay) before electrophoresis.

Polyacrylamide gel electrophoresis (SDS-PAGE) was run in Tricine buffer as previously described (Schägger, 2006). After run, proteins were transferred onto a nitrocellulose membrane for western analysis. The nitrocellulose membrane was first incubated with a blocking solution containing 3% non-fat dry milk (Bio-Rad) in Tris-HCl 10 mM, NaCl 150 mM, Tween 0.05% (TTBS) for 1 h and then the antibody anti-ESP1 (generous gift from Dr. Touhara) was added at a dilution of 1:20,000. After an overnight incubation, the membrane was washed for 1 h with TTBS and an HPR-conjugated secondary antibody (1:1000 in TTBS) was added for 3 h at room temperature. After washing, the membrane was developed with Super Signal West Pico Chemioluminescent Substrate according to the manufacturer’s instruction (Pierce). Marker proteins (EzWay Protein-Blue marker, Komabiotech) were also run and transferred to identify the appropriate size of the immunoreactive product. Films were scanned and the immunoreactive bands were selected and subjected to densitometric analysis.

### RT-PCR

RNA was extracted from fresh tissue and purified using Trizol reagent (Invitrogen Milano, Italy). About 2 μg of total DNAse treated RNA served as template for oligo-dT primed first strand cDNA synthesis with Im-Prom-II Reverse Transcriptase (Promega, Milano, Italy).

Specific PCR primers were designed to amplify ESP1: 5′-TGTTTGCCATGAAATAACATGTGC-3′, 5′-CTTGGTGAATTAGACAAGTTGGTT-3′. The expected amplified sequence encompasses exon 2 between base 306 and 830 of the coding sequence.

PCR was performed in Mastercycler Personal (Eppendorf, Milano, Italy) using AmpliBiotherm DNA polymerase, 3 mM MgCl_2_, 0.2 mM for each dNTPs and 200 pmol forward/reverse target-specific oligonucleotide primers. Cycling parameters consisted of an initial denaturation step (95°C, 2 min) followed by 35 cycles, each of these included a denaturation (95°C, 30 s), a primer annealing (53°C, 30 s), and an extension (72°C, 30 s) step. Reaction was completed by a final extension step at 72°C for 5 min. Semiquantitative analysis of RNA expression was performed on agarose gel after electrophoresis using the NIS-Elements Advanced Research software (Nikon, Firenze, Italy). Bands were excised and subcloned in pGEM-T vector and the plasmidic DNA was subjected to confirmatory sequencing.

## Results

**Figure 1:**
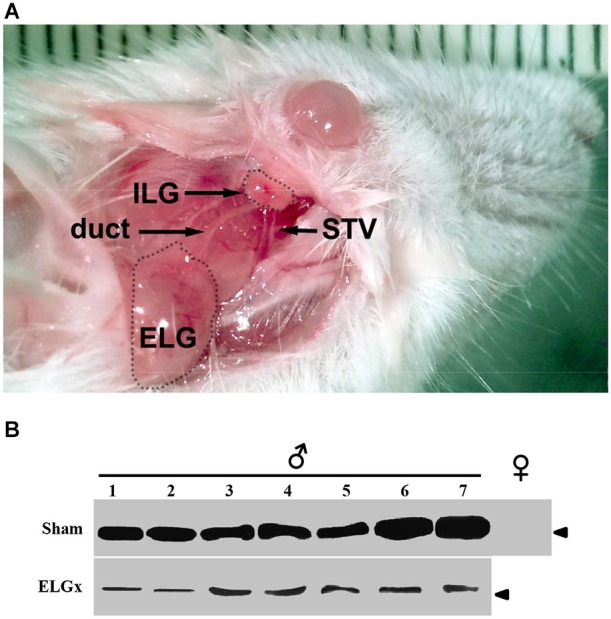
**ESP1-immunoreactivity in the tear fluid after removal of the extraorbital lacrimal glands. (A)** The surgical area after the incision of the skin in the temporal region of the mouse is photographed. Shown are the extraorbital gland (ELG), the intraorbital gland (ILG), the lacrimal duct and the superficial temporal vein (STV). **(B)** The lacrimal fluid of seven BALB/c male mice, which underwent the removal of ELGs (ELGx), and of seven sham operated mice (control) was individually collected and analyzed by western blotting with an antibody against ESP1. Tear fluids were also pooled from five BALB/c female mice and used as a negative control. Arrowhead indicates the position of the molecular marker (aprotinin, 6.5 KDa).

To investigate if the lack of ESP1 expression in BALB/c male mice caused a reduction of the lordosis behavior in receptive females of the same strain, we identified and bilaterally excised the extraorbital glands (ELGs) which are reportedly known to produce this peptide. ELGs are pair structures that lie upon the masseter muscles orally to the parotid glands (Figure 1A). The lacrimal fluid produced by ELG is drained by a lacrimal duct that runs orally to open into the conjunctival sac in proximity to the lateral palpebral commissure. Although the removal of ELG always appeared complete, to make sure that any residual glandular tissues would not contribute in the ESP1 production, the duct was also closed with non absorbable suture. Control mice were also anesthetized and the surgical plan exposed before suturing. After 2 weeks of recovery, the lacrimal fluid of both operated (ELGx) and sham-operated (control) BALB/c mice was individually collected for 2 weeks and samples were run in a Tricine-SDS-PAGE for western analysis.

**Figure 2:**
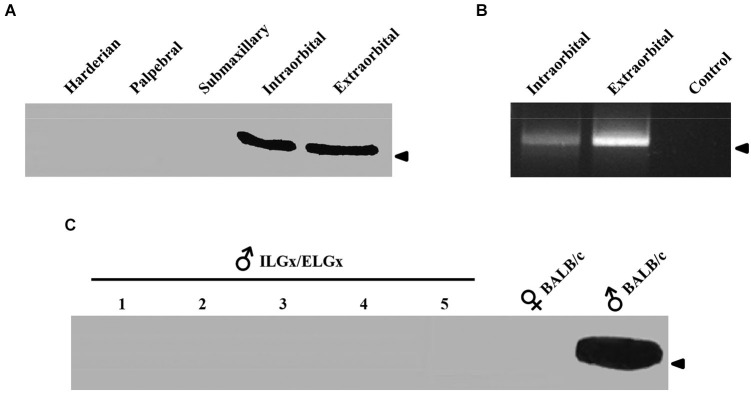
**ESP1 expression in lacrimal glands and tear fluid. (A)** Soluble lacrimal gland extracts of BALB/c mice analyzed for ESP1 immunoreactivity by western blotting. Arrowhead indicates the position of the molecular marker (aprotinin, 6.5 KDa). **(B)** Expression pattern of the ESP1 gene in different lacrimal glands of BALB/c males, as determined by RT–PCR. PCR control without cDNA is also shown. Arrowhead indicates the position of the 500 bp DNA marker. **(C)** ESP1 immunoreactivity in tear fluids, analyzed by western blotting, in five BALB/c males after the removal of both ELGs and ILGs (ELGx/ILGx). Tear fluids of positive (BALB/c males) and negative (BALB/c females) controls were also tested for ESP1 immunoreactivity. Arrowhead indicates the position of the molecular marker (aprotinin, 6.5 KDa).

The immunological analysis with an antibody specific to ESP1 revealed that all ELGx mice still produced lower but detectable levels of ESP1 compared to controls (Figure 1B). This unexpected result led us to investigate the origin of residual production of ESP1 collected in the lacrimal fluid. Thus, we isolated all the lacrimal glands draining into the conjunctival sac and searched for ESP1 expression. The soluble extract of the glands was loaded onto a PAGE gel electrophoresis and the western blot analyzed for immunoreactivity against ESP1. As shown in Figure 2A, the ILGs were also found to produce ESP1. By densitometric analysis, we estimated a 80–100 times reduction of the ESP1 concentration in the lacrimal fluid of ELGx mice. Interestingly, ILGs share the same embryological origin as ELGs and their fluid drains into the ELG lacrimal ducts (Makarenkova et al., 2000; Figure 1A). To prove that ILGs produce ESP1 and to unequivocally exclude any cross reactivity with the anti-ESP1 antibody, ILG total RNA from BALB/c male mice was isolated and reverse-transcribed. RT-PCR of ILG cDNA with ESP1 specific primers indeed confirms that ESP1 is expressed by these glands that in turn are responsible for the remaining immunoreactivity in the tear fluid after removal of ELGs (Figure 2B). Thus, in order to completely abolish the ESP1 production in the BALB/c male mice, both ELGs and ILGs were removed. After ELG and ILG surgery, the tear fluid of individual BALB/c male mice was tested by western blotting for ESP1 immunoreactivity. The removal of both ELGs and ILGs (ELGx/ILGx) resulted in the complete loss of detectable levels of ESP1 in the tear fluid (Figure 2C). Moreover, ELGx and ILGx/ILGx male mice did not show any evident change in stress-related and locomotor behavior that might have been caused by surgery (Table 1). Therefore, we were able to investigate the effects of the orbital glands removal on sexual behavior in mice of the same strain (BALB/c).

**Table 1:** **Locomotor activity measured by the *Emergence Test* in BALB/c male mice after the bilateral removal of both the extra and intra orbital glands (ILGx/ELGx) (*n* = 7 for each group, Time trial, 300 s)**.

Locomotor activity	Sham	ELGx/ILGx	
**Number of walks out**	8 ± 1.8	7.4 ± 1	*F* = 1.7; *p* = 0.79
**Latency (s)**	9.4 ± 1.7	3.8 ± 1.2	*F* = 1.51; *p* = 0.051
**Time outside the box (s)**	235.9 ± 14.8	246.6 ± 8.8	*F* = 1.56; *p* = 0.55
**Rearing (vertical exploration) (s)**	26 ± 6.7	27.8 ± 4.4	*F* = 1.37; *p* = 0.83
**Walking (horizontal exploration) (s)**	110.1 ± 4.5	109.9 ± 7.6	*F* = 0.94; *p* = 0.98
**Self-grooming (s)**	2.7 ± 1.2	2.3 ± 1.8	*F* = 0.25; *p* = 0.87

**Figure 3:**
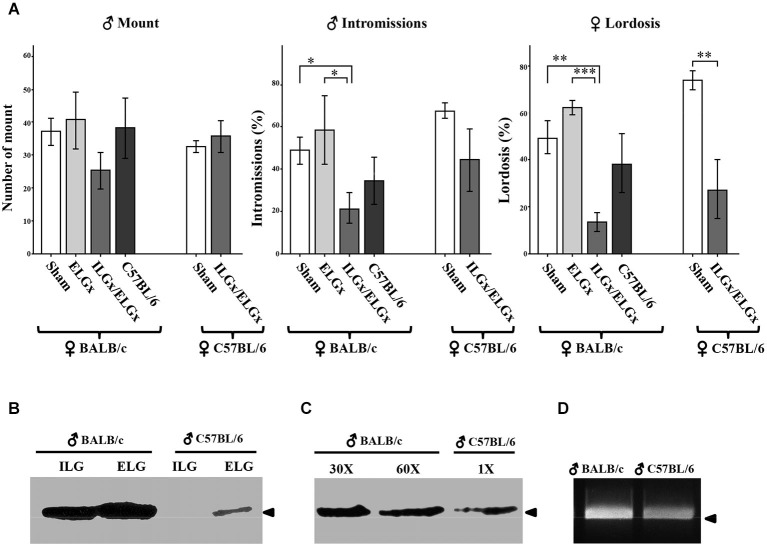
**Removal of the ESP1-expressing lacrimal glands impairs sexual behavior in BALB/c females. (A)** BALB/c and C57BL/6 females were exposed to BALB/c males, which were deprived of the extraorbital glands (ELGx) or both the extraorbital and intraorbital glands (ELGx/ILGx), and to sham operated males (control). Histograms represent the number of male mounts, percentage of successful male intromissions per total mounts and percentage of female lordosis response per total mounts. Error bars represent SE; *n* = 7 for each animal group. * *P* < 0.05, ** *P* < 0.01, *** *P* < 0.0001. **(B)** ESP1 immunoreactivity in the extra and intraorbital gland soluble extracts of BALB/c and C57BL/6 male mice. **(C)** ESP1 immunoreactivity in the tear fluid of BALB/c and C57BL/6 male mice. BALB/c lacrimal fluid was diluted 30 and 60 times, respectively and the same volume of solution was loaded. Arrowhead indicates the position of the molecular marker (aprotinin, 6.5 KDa). **(D)** RT-PCR with ESP1 specific primers of cDNA synthesized from the mRNA of the extraorbital glands of BALB/c and C57BL/6 males. Arrowhead indicates the position of the 500 bp DNA marker.

Sexually naive BALB/c female mice were first ovariectomized and, after a recovery period, they were treated with estrogens and progesterone to induce estrous. When a BALB/c stud male (either control or operated) was introduced into a cage housing the receptive female, the male showed characteristic mounting behavior towards the female that includes mounting and penetration. Similarly, the oestrus female also showed a distinctive behavior which consisted in a reflexive posture upon male mounting. The number of mounting events and penetrations as well as female lordosis behavior was evaluated in a 30 min trial (Figure 3A).

The number of mounts carried out by ELGx, ELGx/ILGx and control mice (*n* = 7 for each group) on BALB/c females did not show any significant difference (*F* = 2.36, σ = 0.16, *p* = 0.685; *F* = 0.57, σ = 0.47, *p* = 0.123 respectively). The percentage of successful intromissions (out of the total mounts) of ELGx/ILGx males (21.9 ± 7.5 SE) towards receptive females was lower than in controls (49.2 ± 6.3 SE) and ELGx males (60.5 ± 15.4 SE) (*F* = 011, σ = 0.75, *p* = 0.021; *F* = 1.09, σ = 0.33, *p* = 0.047, respectively). We then analyzed the sexual behavior of BALB/c females in the same trial. BALB/c females introduced to an ELGx/ILGx male showed a threefold decrease in the percentage of lordosis response (out of the number of mounts) (13.9 ± 4.3 SE) compared to both ELGx (64.4 ± 3.5 SE) and control mice (48.7 ± 6.9 SE) (*F* = 0.65, σ = 0.45, *p* < 0.0001; *F* = 0.64, σ = 0.45, *p* = 0.003 respectively). No significant difference was observed in the lordosis behavior of BALB/c females challenged with either ELGx or control male mice (*F* = 1.21, σ = 0.30, *p* = 0.12).

To investigate whether the removal of ELGs and ILGs produced a similar behavioral response in a female of a different mouse strain, we challenged C57BL/6 receptive females with ELGx/ILGx or BALB/c control mice (*n* = 7 for each group). As for BALB/c females, we found a significant reduction (but not abolishment) of the lordosis behavior of C57BL/6 females when mated to ELGx/ILGx mice (27.1 ± 14.7 SE) compared to controls (73.4 ± 4.5 SE) (*F* = 11.6, σ = 0.08, *p* = 0.01). In contrast, the percentage of intromissions (*F* = 8.37; *p* = 0.14) was not statistically significant as it was the case for the number of mounts (*F* = 6.4, σ = 0.18, *p* = 0.35).

To verify the reproducibility of our test with that of Haga et al. (2010), we wanted to assess if (sham operated) C57BL/6 males, that reportedly lack ESP1 expression, elicited the same behavioral responses as ELGx/ILGx on BALB/c females. Unexpectedly, we found no significant differences in both intromission (*F* = 4.64, σ = 0.06, *p* = 0.27) and lordosis (*F* = 4.2, σ = 0,07, *p* = 0.46) response of C57BL/6 males compared to controls (*n* = 7 for each group) (Figure 3A). Thus, we ask whether this result was due to behavioral differences related to the mouse strain which was employed (C57BL/6 vs. BALB/c) or whether our C57BL/6 males were able to produce ESP1. To answer this question we collected the tear fluid and the ELG-ILG soluble extracts of C57BL/6 males and tested them by SDS-PAGE and western analysis. Although at much lower concentration than in BALB/c mice, we detected ESP1 immunoreactivity in both the tear fluid and the glandular extract of our C57BL/6 males (Figures 3B,C). A densitometric analysis of the immunoreactive signals revealed that the concentration of ESP1 in the tear fluid of C57BL/6 males was approximately 90 times lower than in BALB/c and was comparable to that observed in ELGx males (not shown). This observation was confirmed by RT-PCR on reverse-transcribed mRNA extracted from ELGs and ILGs and amplified with ESP1-specific primers (Figure 3D).

## Discussion

In this work, we have analyzed the behavioral responses of oestrus BALB/c females elicited by BALB/c male mice that were surgically deprived of the extraorbital and intraorbital lacrimal glands that produce ESP1 (Kimoto et al., 2005). This peptide was shown to play a pheromonal role in the sexual communication of the mouse (Haga et al., 2010) since it was demonstrated that BALB/c mice that produce and secrete ESP1 into the conjunctival sac enhances lordosis behavior in receptive C57BL/6 females. In contrast, C57BL/6 males, that reportedly do not express ESP1, fail to induce such behavior in BALB/c females (Haga et al., 2010).

Our aim was to investigate if this behavioral response was independent of the mouse strain which is employed and whether ELGs and ILGs are indeed important in mediating pheromonal communication.

In preliminary observations to this study, we found that ESP1, which was thought to be exclusively expressed in the extraorbital gland, is also produced by the ILGs. This pair glandular structure lies close to the lateral palpebral commissure and drains the fluid into the same duct as the extraorbital glands with which they share the same embryological origin (Makarenkova et al., 2000). Mice that underwent the bilateral surgery of ELGs and ILGs did not show any ESP1 immunoreactivity in the tear fluid whereas the removal of the ELGs alone did not abolish ESP secretion into the conjunctival sac.

In a behavioral context, BALB/c mice with the surgical ablation of both ELG and ILG (ELGx-ILGx) failed to enhance the lordosis behavior in BALB/c females confirming that this effect is not dependent on the strain which was employed (C57BL/6 or BALB/c). This is also confirmed by experiments where C57BL/6 females were paired with ELGx-ILGx males in a lordosis behavioral test. The surgical removal of the ELG alone in BALB/c mice (ELGx) was not sufficient to decrease lordosis behavior in BALB/c females. In fact, ELGx mice produce small but detectable amounts of ESP, thus suggesting that females are able to sense and respond to very low concentrations of ESP1 via the vomeronasal organ. This is consistent with what is observed in C57BL/6 males that also succeeded in enhancing lordosis behavior in BALB/c females. In C57BL/6 mice, we found levels of ESP1 in the tear fluid which were comparable to that observed in ELGx mice. Nevertheless, this result was unexpected as it disagrees with previous studies where C57BL/6 males were reportedly unable to produce ESP1 and therefore to enhance lordosis behavior in females (Haga et al., 2010). Thus, it is possible that variability in the ESP1 expression within the same strain may exist. This may be due to small genetic variations or caused by the different environments where these animals live. Overall, our results highlight the pheromonal role of the lacrimal glands and established that, ESP1, probably along with other pheromones, acts as a modulator of female lordosis behavior via the vomeronasal system. Lordosis, thus, appears to be a sexual performance largely, although not exclusively, modulated by ESP1 stimulation through the activation of the vomeronasal system. The modulation of lacrimal gland secretion on female behavior appears effective across different mouse strains providing they express, even at very low level, this peptide. This suggests that the neural pathways underlying the transmission of the lacrimal pheromone signal are preserved also in mouse strains that do not constitutively express ESP1. It is worth noting that the removal of ELGs and ILGs may also abolish the production of additional pheromones that are involved in other behavioral performances. The persistence of lordosis in mice deprived of both the extra and ILGs (see Figure 3A) also establishes that this sexual performance is only partially dependent on the secretion of these glands and that other chemical or physical stimuli are involved. Thus, the animal model which we have developed may be interesting to elucidate different aspects of the pheromonal communication in rodents. For example it was reportedly observed that ESP1 stimulation in BALB/c but not in C57BL/6 males does not induce cFos expression in the vomeronasal and accessory olfactory bulb neurons, as a result of desensitization caused by self-secreted ESP1(Haga et al., 2010). In this context, it might be interesting to investigate if desensitization is strain-specific (irreversible) or cFos responsiveness can be restored after lacrimal gland removal. Furthermore, ELGx/ILGx mice could be also successfully employed to investigate the behavioral responses in mice with the genetic ablation of the vomeronasal transduction molecules (Stowers et al., 2002; Norlin et al., 2003; Montani et al., 2013; Oboti et al., 2014).

## Author contribution

Rosa Maria Cavaliere performed the experiments, collected and analyzed the data. Filippo Ghirardi performed the initial experiments and Roberto Tirindelli wrote the manuscript and directed the project.

## Conflict of interest statement

The authors declare that the research was conducted in the absence of any commercial or financial relationships that could be construed as a potential conflict of interest.
